# Corneal biomechanics and diagnostics: a review﻿

**DOI:** 10.1007/s10792-024-03057-1

**Published:** 2024-03-13

**Authors:** Maria Angeliki Komninou, Theo G. Seiler, Volker Enzmann

**Affiliations:** 1 Department of Ophthalmology, Bern University Hospital Inselspital, University of Bern, Bern, Switzerland; 2 Present Address: Institute of Intensive Care Medicine, University Hospital Zurich & University of Zurich, Zurich, Switzerland; 3 Klinik Für Augenheilkunde, Universitätsklinikum Düsseldorf, Düsseldorf, Germany; 4 Institut Für Refraktive Und Opthalmo-Chirurgie (IROC), Zurich, Switzerland; 5 Wellman Center for Photomedicine, Massachusetts General Hospital, Harvard Medical School, Boston, MA USA; 6 Department of BioMedical Research, University of Bern, Bern, Switzerland

**Keywords:** Cornea, Biomechanics, Biomechanical properties, Brillouin spectroscopy

## Abstract

**Purpose:**

Corneal biomechanics is an emerging field and the interest into physical and biological interrelations in the anterior part of the eye has significantly increased during the past years. There are many factors that determine corneal biomechanics such as hormonal fluctuations, hydration and environmental factors. Other factors that can affect the corneas are the age, the intraocular pressure and the central corneal thickness. The purpose of this review is to evaluate the factors affecting corneal biomechanics and the recent advancements in non-destructive, in vivo measurement techniques for early detection and improved management of corneal diseases.

**Methods:**

Until recently, corneal biomechanics could not be directly assessed in humans and were instead inferred from geometrical cornea analysis and ex vivo biomechanical testing. The current research has made strides in studying and creating non-destructive and contactless techniques to measure the biomechanical properties of the cornea in vivo.

**Results:**

Research has indicated that altered corneal biomechanics contribute to diseases such as keratoconus and glaucoma. The identification of pathological corneas through the new measurement techniques is imperative for preventing postoperative complications.

**Conclusions:**

Identification of pathological corneas is crucial for the prevention of postoperative complications. Therefore, a better understanding of corneal biomechanics will lead to earlier diagnosis of ectatic disorders, improve current refractive surgeries and allow for a better postoperative treatment.

## Background

Corneal biomechanics refers to the mechanical properties and behavior of the cornea, which is the clear, dome-shaped front surface of the eye. It involves the study of how the cornea responds to applied forces, deforms, and recovers its shape [1, 2]. The corneal tissue is composed of five basic layers. The cornea’s outermost layer is the epithelium, comprising around 10 percent of the whole tissue’s thickness [3]. It blocks the entrance of foreign materials like water, dust and bacteria into the other layers of the cornea but also the whole eye. Moreover, it provides an ideal surface that absorbs cell nutrients and oxygen and distributes them to the rest of the cornea but is also filled with nerve endings making it sensitive to rubbing or scratching [4]. Basement membrane is the part of the epithelium where the epithelial cells are organized. Below the basement membrane, a transparent sheet of tissue named Bowman’s layer is situated. It forms scars when healing after an injury. However, when the formed scars are centrally located and/or large they can lead to vision loss [5]. Beneath Bowman’s layer the stroma is located, comprising 90 percent of cornea’s thickness [6]. It consists of collagen, that provides the strength, form and elasticity to the cornea, and water [7], but does not contain any blood vessels [8]. In detail, it is composed of dense, regularly packed collagen fibrils shaped in orthogonal layers or lamellae. Limbal epithelial stem cells (LESCs) located in the limbus at the corneoscleral junction are responsible for regenerating and renewing the corneal epithelium [9]. The Descemet’s membrane can be considered as the basement membrane of corneal endothelium [10], helps keep the endothelial monolayer in place to maintain corneal clarity [11] and is located under the stroma [12]. It accounts for the stiffest layer of the cornea and is, thus, critical for corneal integrity, which contributes in protecting the eye from penetrating injury and in guaranteeing its refractive function [13]. It is composed of collagen fibers and therefore acts as a natural barrier against microorganisms and trauma [14]. The innermost part of the cornea is the endothelium. It is an extremely thin layer that pumps the excess fluid leaking slowly from inside the eye to the stroma, keeping the balance between fluid moving to and from the cornea. The cornea is thinner at the center, presenting a gradual increase towards the periphery [15, 16]. Given the fact that the cornea is smooth and transparent, but also strong and durable, it supports the eyes in two ways. It helps to shield the eye from germs, dust, and other harmful matter by sharing this task with the eyelids, the eye socket, tears, and the white part of the eye (sclera) but it also acts as the eye's outermost lens. It functions like a window that controls the entry of light into the eye. The biomechanical properties that characterize the cornea play an important role in maintaining its shape and transparency [17]. These properties are dependent on biochemical and physical components such hydration, elasticity, viscosity, and the thickness of corneal stroma [18].

## Basic biomechanical concepts

It is important to be aware of the meaning of the basic corneal properties such as elastic and viscous response, stiffness and hysteresis in order to better understand the results of corneal biomechanical evaluations. Therefore, here is the general definition of the main terms in this regard:

Biomechanical stress is a reaction of any material under a load that tries to separate atoms within a solid. Assuming that the load is uniformly distributed within a homogeneous material the sum of all these stresses (*σ*) must be equal with the force *F* (either tension or compression) acting perpendicular to an imaginary plane surface passing through a piece of material divided by the cross-section area (*A*) in N/m^2^ [19].

1 $$\sigma = F/A,$$

In order to characterize the magnitude of deformation in response to stress another property called strain (*ε*) is defined:

2 $$\varepsilon = \Delta L/L,$$

Typically, it is the fractional amount of elongation (increase in length) or contraction (decrease in length) in a material caused by a stress.

The elastic response of a material is attributed to the instantaneous and reversible deformation under an external load therefore an elastic material has a linear relationship between stress and strain [20]. The constant of proportionality between stress and strain is known as elastic modulus, also called *Young’s modulus (E),* defined as the ratio of the *stress* (load per unit area) and the *strain* (deformation/displacement per unit length) [21].

3 $$E = \sigma /\varepsilon ,$$

Note that since ε is dimensionless, E also has units of s, that is, force per unit area. Being measured *in vitro,* corneal Young’s modulus varies from 0.1 to 57 MPa, due to variations in testing conditions and methods used [22–24]. The higher the Young’s modulus, the stiffer the tissue leading to lesser deformation and faster recovery.

However, there is another category of materials, including almost all biological materials and polymer plastics, that displays gradual deformation and recovery when they are subjected to loading and unloading [25]. The response of this kind of materials is dependent upon how quickly the load is applied or removed. This time-dependent behavior is called *viscoelasticity*. A viscoelastic material possesses both fluid and solid properties since viscosity, a fluid property, is considered as measure of resistance to flow and elasticity is a solid material property [26].

Finally, c*orneal hysteresis (CH)* can be described as the portion of input energy dissipated during mechanical strain due to viscosity of the corneal tissue and *corneal resistance factor (CRF)* is a measurement of corneal resistance that is relatively independent of intraocular pressure (IOP) that is useful diagnosis and prognosis after refractive surgery [27–29].

## Factors determining corneal biomechanical properties

### Extracellular matrix components

In the biology field, the extracellular matrix (ECM) is a three-dimensional (3D) network of extracellular macromolecules including glycoproteins, collagen and enzymes, providing biochemical and structural support to the surrounding cells [30, 31]. In corneal biomechanics, it plays an important role in the function of the corneal epithelium (development, growth, differentiation, and migration). The right assembly of the ECM is crucial for corneal function since it regulates transparency, shape, avascularity and wound healing, as well as for mechanical stability required for corneal shape and curve [32–34]. Corneal transparency is the major refractive element of the eye that is caused by regular arrangement of collagen fibers and interfibrillar space, which appears in the cornea during embryonic development [35].

Collagen fibrillogenesis starts with the interactions between molecules, matrix components and keratocytes [8]. Collagen V regulates the nucleation of protofibril assembly in the stroma [36]. It is also enriched in small leucine-rich proteoglycans (SLRPs) that regulate linear and lateral collagen fibril growth. Collagen XII and XIV, also known as fibril-associated collagens (FACITs) play an important role in the regulation of inter-lamellar interactions and fibril packing [37].

### Hydration

Hydration not only affects corneal transparency, but also its elastic modulus. The more hydrated the corneal tissue, the lower the elastic modulus. It potentially arises from an altered collagen attachment to the proteoglycans and/or glycosaminoglycans based on their ionic interaction [38, 39].

### Environmental factors

The influence of environmental factors on corneal biomechanics has been poorly investigated. However, it is already known that mechanical disturbance (e.g., eye rubbing) is strongly associated to keratoconus [40]. Another study showed higher CH values in smokers than in non-smokers [41]. Furthermore, exposure to a higher ambient UV radiation over a 65-year period decreased CH values, indicating a viscoelastic change of the cornea [42].

### Hormonal fluctuations

Fluctuations of estrogen levels in blood through the menstrual cycle and pregnancy lead to changes and specifically increase in corneal thickness, decreases corneal hysteresis and corneal resistance factor [43–45]. Furthermore, pregnancy and disturbed levels of thyroid hormones are reported in corneal ectasia, a pathologic forward bulging and thinning of the cornea [46]. Estrogen administration has been shown to reduce biomechanical stiffness in *ex vivo* corneas [47].

## Factors leading to alterations of biomechanical properties

### Age

It is widely known that biomechanical properties of the cornea vary with age. For instance, young age is a risk factor for both keratoconus progression and iatrogenic ectasia whereas the incidence of keratoconus decreases with age [48–50]. Many human tissues, usually becoming less flexible by age. It has been demonstrated that the main changes refer to structure, composition, and mechanical properties [51]. In general, corneal stiffness increases because of gain in ocular rigidity coefficient, Young's modulus and cohesive tensile strength [52–54], while decreasing in viscous behavior like hysteresis decreases with age [55].

### Intraocular pressure (IOP)

Intraocular pressure is the fluid pressure of the eye and accounts for a measure of force per area. IOP is a measurement including the magnitude of the force exerted by the aqueous humor on the internal surface area of the anterior eye [56]. IOP is considered as the key parameter to determine the health of the eye since it could be increased due to anatomical problems, genetic factors, inflammation of the eye, or as a side-effect from medication [57] but is also a major risk factor for glaucoma [58]. The normal intraocular pressure is varies between 12 and 22 mm Hg, with cyclic fluctuations of 2–3 mm Hg throughout the day with the lowest being at night due to circadian regulation of aqueous humor secretion [59] and peaking in the early morning [60]. Levels above 22 mm Hg are considered pathologic [61].

### Contact lens wear

While soft contact lenses and scleral lenses are effective tools for visual correction, they could potentially both negatively impact corneal biomechanics in several ways. Soft contact lenses can inhibit the oxygen supply to the cornea, leading to corneal edema or swelling and changes in corneal thickness [61]. Long-term use may also cause corneal warpage or remodeling, leading to increased corneal surface asymmetry and irregularity. On the other hand, scleral lenses, due to their large size and rigid design, can potentially cause hypoxia and physiological changes in the cornea and underlying tissues [62, 63]. The lens-cornea clearance, along with lens fitting and wearing time, may induce stress and potential changes to the corneal structure. Additionally, the pressure exerted by these lenses on the sclera may influence intraocular pressure, thereby affecting corneal biomechanics. Therefore, although both types of lenses have their unique benefits, their potential effects on corneal biomechanics necessitate regular ophthalmological examinations for lens wearers [64].

### Central corneal thickness (CCT)

Central corneal thickness is considered as a biometric entity [65, 66], and can be altered by age, ethnicity or genetics [67] but also experiences circadian changes. It is affected by prostaglandins, topical dorzolamide, topical beta-blockers, estrogen levels during ovulation and pregnancy and diabetes [68]. It is a measure of tissue mass and a possible estimator for corneal rigidity and is correlated to biomechanics and an important parameter for the assessment of different ocular diseases such as glaucoma [69]. Corneal thickness can affect the measurements of CH and CRF due to the mechanical properties of the cornea. Thicker corneas generally tend to have higher CH and CRF values, while thinner corneas typically have lower values. This relationship is because corneal thickness is related to corneal rigidity and its ability to absorb and dissipate energy. Thicker corneas have a greater structural integrity and stiffness, which leads to a higher resistance to deformation. As a result, thicker corneas exhibit higher CH and CRF values. Conversely, thinner corneas have a lower capacity to absorb and dissipate energy, resulting in lower CH and CRF values [70, 71].

## Tools for evaluation of corneal biomechanics

As mentioned above, evaluation of corneal biomechanics is crucial in order to assess changes in the corneal tissue. Up to date, several techniques have been developed and used in an attempt to quantify the biomechanical properties of the cornea. These techniques can be divided into two groups. The first one is *ex vivo* destructive testing and the second one is *in vivo* non-destructive testing [72, 73] (Table 1).

**Table 1:** Summary of methods used ex-vivo & in-vivo for corneal biomechanics assessment

Technique	In-vivo/ex-vivo	Measurement variables	Detection of biomechanical changes
Strip extensometry	Ex-vivo	Elastic moduli	Stress–strain behavior of cornea
Inflation tests	Ex-vivo	IOP change	Corneal stiffness after CXL
Speckle pattern interferometry	Ex-vivo	Corneal displacement	Corneal deformation, IOP detection
ORA	in-vivo	Cross-sectional spatial and dynamic deformation	Parameters for detecting sub clinical keratoconus
CORVIS ST	In-vivo	Captures cornea deformation	Detects ectatic diseases
Goldmann applanation tonometry	In-vivo	Changing the applanating force	IOP detection
Brillouin spectroscopy	In-vivo	3D Brillouin modulus	Brillouin modulus changes after CXL and in keratoconus

### Ex vivo destructive testing

#### Strip extensometry

Strip testing (also known as coupon testing) is the most common *ex vivo* technique used experimentally to determine the stress-strain behavior of corneas. The lack of human tissue specimens made it necessary in some cases to rely on animal models, primarily porcine and rabbit tissue [74]. When measuring—a strip of corneal tissue with a specific width is dissected and attached to the grips of a slow rate tension machine while monitoring its behavior under stress. The applied stress on the tissue is plotted against the strain to derive Young's modulus [75].

*Limitations*: the variation in sample length leads to a non-uniform stress distribution across its width. The flattening of the originally curved specimen produces tensile and compressive strains on the posterior and anterior sides respectively, could be considerable even though the CT might be small in relation to the other dimensions [74].

#### Inflation tests

Inflation test measures the biomechanical properties by expanding the entire cornea, based on IOP change, while keeping the integrity of the tissue. This testing has been successfully applied to detect the increase of corneal stiffness after collagen cross-linking, a minimally invasive technique to treat progressive keratoconus [76] and has also been used to demonstrate that corneal behavior is affected by age and test loading rate. However, it lacks the ability to monitor the different expansions of the cornea at different locations [77] and the results vary between using an artificial anterior chamber or whole globe mounts [78].

#### Speckle pattern interferometry

Electronic Speckle Pattern Interferometry (ESPI) and Radial Shearing Speckle Pattern Interferometry (RSSPI) are both optical techniques used in corneal biomechanics to measure the deformation and mechanical properties of the cornea.

- ESPI is an interferometric technique that utilizes laser light to measure the deformation of an object's surface. In corneal biomechanics, ESPI can be used to assess the response of the cornea to external stimuli or forces. The basic principle involves splitting a laser beam into two separate beams. One beam is directed towards the cornea, while the other is directed towards a reference mirror [79]. The two beams are then recombined, creating an interference pattern on a detector. As the cornea deforms, the interference pattern changes, allowing for the measurement of corneal displacement and strain. ESPI has been used in corneal biomechanics studies to investigate the effects of intraocular pressure, external loading, and surgical procedures on corneal deformation. By analyzing the interference patterns, researchers can quantify corneal stiffness, elasticity, and other mechanical properties [80].
- RSSPI is another interferometric technique used in corneal biomechanics research. It is based on the principle of shearing interferometry, where a shearing plate is used to introduce a known displacement gradient between two points on the cornea [81]. This displacement gradient results in a shearing of the speckle pattern created by the laser light reflected from the cornea. By analyzing the changes in the sheared speckle pattern, researchers can determine corneal displacements and strain.

*Limitations*: ESPI and RSSPI are valuable techniques for studying corneal biomechanics but have limitations. They are sensitive to environmental conditions, requiring controlled environments to ensure accuracy. Both techniques have a limited field of view, hindering comprehensive corneal deformation analysis and the data analysis process is complex and time-consuming, limiting real-time applicability. Additionally, the invasive nature of applying coatings or markers on the cornea can affect natural corneal behavior.

### In vivo non-destructive testing

Several non-destructive methods have also been developed and tested for measuring corneal biomechanics in vivo, minimizing the effect of removing the cornea from its native environment. This opens the possibility of different clinical applications of these devices. However, there is a limitation when it comes to the scope of investigation since all measurements have to be performed without affecting the function of the eye. The current available devices include Ocular Response Analyzer (ORA), Corvis ST, Tonometry and Dynamic corneal imaging using Placido, Scheimpflug, or optical coherence tomography devices. Some other devices that have been used in research but are not widely introduced in clinics include Brillouin spectroscopy and supersonic shear imaging surface wave elastometry [80, 82, 83].

#### Ocular response analyzer (ORA)

The ocular response analyzer (ORA; Reichert Technologies, Depew, NY), a newer type of tonometer, is a non-invasive device that analyses corneal biomechanical properties fast and effective. ORA main function is based on the principles of non-contact tonometry, in which the IOP is determined by the air pressure required to applanate the central cornea. As described by Lau and Pye [84] an air pulse of increasing force lasting approximately 20 ms is directed onto the eye, leading to progressive corneal deformation. The deformation is recorded by using infrared corneal reflex [85]. The measured values are:

1. the IOPcc: Corneal compensated IOP, which is less affected by corneal thickness and properties and can be empirically determined by linear combination of applanation pressure 1 (P1) and pressure 2 (P2),
2. the IOPg or Goldmann correlated IOP, which is analogous to Goldmann Tonometry and can be considered as the average of applanation P1 and P2,
3. the corneal hysteresis (CH), which is the viscoelastic response (difference of P1 and P2) and corneal resistance factor (CRF), viscoelastic response weighted for central corneal thickness (empirically determined linear combination of P1 and P2) but also.

It is used by surgeons to detect corneal diseases such as keratoconus, to predict the risk of ectasia after refractive surgeries and glaucoma progression as CH of glaucomatous eyes is lower than that of their healthy counterparts [86].

Limitations: Deviations from the expected results between CH and CRF have been observed. ORA analysis is not adequate to analyze the cornea after cross-linking and biomechanical modeling is usually needed. Further exploration of the morphology of the ORA’s corneal signal (Bio-corneagram analysis) could add to the robustness of this technique [87, 88].

#### Oculus CORVIS ST

CORVIS ST non-contact tonometer (OCULUS Optikgeräte GmbH, Wetzlar, Germany) was placed on the global market back in 2010. It provides biomechanical parameters of the cornea [89, 90] and corneal deformation that is influenced by IOP, thickness, and innate biomechanical properties [91]. Furthermore, it allows ectatic diseases such as keratoconus to be detected at a very early stage. In short, the instrument’s camera records a sequence of images, capturing corneal deformation following a rapid air puff. This camera is capable of capturing 4,330 images per second that are analyzed to quantify central corneal thickness (CCT), deformation amplitude (DA), applanation length, corneal velocity and the concave radius of curvature (R) at the point of highest concavity, biomechanical corrected IOP (bIOP), Ambrósio’s Relational Thickness horizontal (ARTh), stiffness parameter at first applanation (SP A1) and Corvis Biomechanical Index (CBI) [92–95].

Limitations: In some diseases, such as keratoconus, lateral motion of the cornea might not be captured by Corvis ST as it records only 2D images of the corneas.

#### Goldmann applanation tonometry

Applanation tonometry is based on the Imbert-Fick principle [96], which indicates that the pressure inside an ideal thin-walled sphere equals the force necessary to flatten the surface divided by the area of flattening (*P* = *F*/*A*, *P* = pressure, *F* = force and *A* = area). With this method, by changing the applanating force, the cornea is flattened and the IOP can be determined. Goldmann applanation tonometry is influenced by corneal properties, including thickness, curvature, and Young’s Modulus [97, 98]. The similar Perkins applanation tonometer yields IOP measurements that are closely comparable with GAT [99].

*Limitations*: the eye under examination has to be numbed with local anesthesia, includes a high level of skill to operate, there is lack of ability to measure in supine patients and decreased accuracy on an irregular or scarred cornea [100].

#### Brillouin spectroscopy

Currently, there is no way to assess corneal biomechanics in humans with high spatial resolution and clinical conclusions can only be drawn by geometrical analysis of the cornea. Most of the current knowledge has been obtained from ex vivo destructive biomechanical testing described above. Brillouin spectroscopy might be a possible way to assess *in vivo* corneal biomechanics. It is an emerging technique based on Brillouin light scattering from acoustic waves providing a non-destructive contactless probe of the mechanics on a microscale. The light photons interact with natural acoustic photons in the cornea and lead to Brillouin shift, which is related to elastic modulus [101–103]. The shift in frequency of the Brillouin spectrum of the scattered light, caused by the photon interaction leading to loss or gain of energy, is related to the elastic modulus (*M*′) of the material, as shown in this equation (*ρ* = mass density, *λ* = wavelength, Ω = frequency shift, and *n* = the refractive index): *M*′ = *ρλ*^2^Ω^2^/4*n*^2^.

*Limitations*: the system is sensitive to temperature, vibration and alignment. The transition into an accurate and reproducible commercially available clinical device is a hurdle that has yet to be overcome [104].

#### Corneal transient elastography (CTE)

Corneal transient elastography is a non-invasive imaging technique that measures corneal stiffness or elasticity by generating and analyzing shear waves in the cornea [105, 106]. It provides information about corneal biomechanics and has potential applications in assessing corneal diseases and monitoring treatment response and had been primarily used in breast tissue imaging [107]. It uses two different types of energy to measure the corneal properties, the generation of a remote palpation in the cornea and ultrafast (20 000 frames/s) ultrasonic images. The shear wave propagation that is created is linked to local elasticity. With this technique, high-resolution images can be achieved.

### Biomechanical models

Biomechanical models are being developed and tested at multiple clinical sites. These models use mathematical expressions to describe the response of a material to disturbance [108]. Finite elements is the best known method for modeling and studying complex structures that depends on the availability and quality of the input values. Providing accurate models, improvement in outcomes of corneal surgery and individualized analysis and simulations of refractive surgery for each patient could be achieved [109].

## Clinical applications of corneal biomechanics

As it was mentioned before, corneal biomechanics is a diagnostic modality that could allow the early detection of weaker corneas even at a subclinical stage and pathological conditions in the anterior part of the eye leading to more effective treatments. Clinical applications of biomechanics are envisioned for the following corneal diseases:

### Cataract

Cataract, characterized by clouding of the lens, is a prevalent eye condition affecting the elderly and is a leading cause of global blindness [110–112]. It can result in faded colors, halos around light, and blurry or double vision. Cataract development is primarily associated with age-related changes in the crystalline lens [113]. Other factors, such as malnutrition, exposure to excessive ultraviolet rays, trauma, atopic disorders, diabetes, and congenital disorders, can also contribute to its occurrence [114]. Mild cataract symptoms can be managed with glasses, but in severe cases, the only effective treatment is cataract surgery. During cataract surgery, the cloudy lens is removed and replaced with an artificial one. It is noteworthy that corneal biomechanical changes have been observed following cataract surgery, including alterations in corneal thickness and hysteresis, which can impact intraocular pressure (IOP) measurements and corneal stability post-surgery [115]. Understanding corneal biomechanics is crucial in planning the surgical approach and determining the optimal location for the incision during cataract surgery. By considering corneal biomechanics, surgeons can optimize surgical outcomes and promote corneal stability during the healing process since their proper understanding helps in choosing an incision location that minimizes induced astigmatism and maximizes wound integrity [116–118]. Ensuring a stable and properly sealed incision, promoting optimal visual outcomes and reducing the risk of complications such as corneal edema, wound leakage, or induced corneal irregularities can enhance surgical outcomes, promote faster healing, and improve overall patient satisfaction after cataract surgery.

### Glaucoma

Corneal biomechanics, specifically corneal hysteresis (CH), is a crucial parameter in the diagnosis and management of glaucoma [119]. Glaucoma is a prevalent eye disease that can lead to irreversible blindness if left untreated. Early detection of glaucoma is challenging as initial stages often lack noticeable symptoms. It involves the progressive degeneration of retinal ganglion cells and can manifest as primary or secondary, open-angle or angle-closure glaucoma [120, 121]. Although the underlying mechanisms are not fully understood [122, 123], lower CH has been strongly associated with glaucoma-related structural and functional changes, such as optic nerve damage and visual field loss [124]. Assessing corneal biomechanics, especially CH, provides valuable insights into corneal properties, aiding in glaucoma diagnosis, monitoring disease progression, and making informed treatment decisions. Additionally, corneal structure and thickness, mainly represented by CH, serve as useful parameters for diagnosing and monitoring glaucoma. Eyes with lower CH, particularly in advanced disease cases, exhibit structural weakness, indicating that CH and corneal characteristics can be considered independent risk factors for glaucoma [125].

### Keratoconus

Keratoconus is a progressive eye disease where instead of having a round cornea it turns into a cone-like shape due to thinning. This altered shape refracts light as it enters the eye on its way to the light-sensitive retina, causing distorted vision. Keratoconic eyes show a weaker stress-strain response alongside with a more disorganized collagen network [23, 126], but also reduced CH [127]. Corneal crosslinking (CXL) [128], a minimally invasive technique designed to treat progressive keratoconus, has been introduced into clinical routine to artificially stiffen pathologically weak corneas and prevent a further progression of the disease. This procedure consists of application of riboflavin, a vitamin B, clinically used in humans and its activation by means of UVA-light. The stiffening effect is achieved by generating reactive oxygen species (ROS), which are responsible for the formation of new bonds within the collagen fiber between the proteoglycans. Although very important, the knowledge about the role of oxygen in this process is very limited and needs to be further investigated [129]. Understanding corneal biomechanics plays a crucial role in the comprehensive management of keratoconus. Assessing corneal biomechanics, such as corneal hysteresis, allows clinicians to objectively evaluate the altered mechanical behavior of the cornea in keratoconus [130]. The reduced corneal hysteresis observed in keratoconic eyes indicates a diminished ability to absorb and dissipate energy, contributing to the weakened stress-strain response and progressive corneal thinning. In the context of treatment, corneal crosslinking (CXL) has become a widely used intervention for progressive keratoconus [131]. The success of CXL relies on the creation of additional cross-links between collagen fibers, which helps strengthen the cornea and halt the disease progression. Monitoring corneal biomechanics is essential in assessing the effectiveness of CXL treatment and evaluating the cornea's response to the intervention. Regular assessment of corneal biomechanics allows for long-term monitoring, enabling early detection of corneal destabilization and guiding appropriate management strategies. Overall, incorporating corneal biomechanics into clinical practice enhances personalized care for keratoconus patients [132, 133].

### Iatrogenic corneal ectasia

Iatrogenic corneal ectasia is a rare complication of refractive surgery with an incidence of 0.2–0.66%, but also one of the most feared situations that can occur after corneal laser surgery [134, 135]. The progression of the disease weakens the cornea leading to biomechanical degeneration from delamination and interfibrillar fracture causing severe loss of corrected visual acuity [136]. Corneal biomechanics assessment is crucial since it helps quantify corneal weakening and assess its severity, guiding treatment decisions [137]. Corneal hysteresis reflects the cornea's ability to absorb and dissipate energy, providing valuable insights into corneal mechanical changes. Corneal crosslinking (CXL) is a treatment option that strengthens the cornea by inducing collagen cross-linking, and corneal biomechanics assessment aids in evaluating the effectiveness of CXL and monitoring the cornea's response. In severe cases, where visual impairment is significant, corneal biomechanics assessment assists in identifying the need for more invasive interventions like grafting to restore corneal integrity and improve visual function.

## Conclusions

Corneal biomechanics is a rapidly evolving field with significant implications for the diagnosis and treatment of various corneal diseases. The understanding of corneal biomechanics has advanced through ex vivo biomechanical testing and the development of non-destructive, in vivo measurement techniques. These advancements have provided valuable insights into the mechanical properties of the cornea and their relationship to ocular health. Alterations in corneal biomechanics have been identified as important factors in the development and progression of corneal diseases such as keratoconus and glaucoma. Therefore, the ability to accurately assess corneal biomechanics in vivo has become crucial for early detection, diagnosis, and monitoring of these conditions. Non-destructive techniques such as Ocular Response Analyzer (ORA), Corvis ST, and Brillouin spectroscopy offer promising tools for assessing corneal biomechanical properties in a clinical setting. The extracellular matrix (ECM) components, hydration, environmental factors, hormonal fluctuations, age, intraocular pressure (IOP), contact lens wear, and central corneal thickness (CCT) are among the factors that influence corneal biomechanics. Understanding the role of these factors and their impact on corneal properties can aid in the identification of individuals at risk for corneal diseases and help guide treatment strategies. The clinical applications of corneal biomechanics are wide-ranging. In cataract surgery, knowledge of corneal biomechanics can assist in surgical planning and incision placement to optimize outcomes and promote corneal stability during the healing process. For glaucoma management, corneal biomechanics, particularly corneal hysteresis (CH), provides valuable information for diagnosis, disease progression monitoring, and treatment decision-making. In the case of keratoconus, understanding corneal biomechanics is essential for early detection, prognosis, and treatment planning, such as the application of corneal cross-linking. Additionally, the assessment of corneal biomechanics is crucial in addressing iatrogenic corneal ectasia, a rare but serious complication of refractive surgery. In conclusion, advancements in corneal biomechanics research have paved the way for improved understanding, diagnosis, and treatment of corneal diseases. Further research and technological advancements are needed to refine measurement techniques, develop comprehensive biomechanical models, and explore additional clinical applications. The continued exploration of corneal biomechanics will undoubtedly contribute to earlier disease detection, improved surgical outcomes, and enhanced postoperative care, ultimately leading to better visual health and quality of life for patients.

## Data Availability

Not applicable.
